# The role of social network structure and function in moderate and severe social and emotional loneliness: The Dutch SaNAE study in older adults

**DOI:** 10.1016/j.heliyon.2023.e23734

**Published:** 2023-12-16

**Authors:** Lisanne CJ. Steijvers, Stephanie Brinkhues, Bianca Suanet, Mandy MN. Stijnen, Christian JPA. Hoebe, Nicole HTM. Dukers-Muijrers

**Affiliations:** a Department of Health Promotion, Care and Public Health Research Institute (CAPHRI), Maastricht University, Maastricht, the Netherlands; b Department of Sexual Health, Infectious Diseases and Environmental Health, Living Lab Public Health, South Limburg Public Health Service, Heerlen, the Netherlands; c Department of Knowledge and Innovation, Living Lab Public Health, South Limburg Public Health Service, Heerlen, the Netherlands; d Department of Sociology, Faculty of Social Sciences, Vrije Universiteit Amsterdam, Amsterdam, the Netherlands; e Department of Social Medicine, Care and Public Health Research Institute (CAPHRI), Maastricht University, Maastricht, the Netherlands; f Department of Medical Microbiology, Infectious Diseases and Infection Prevention, Care and Public Health Research Institute (CAPHRI), Maastricht University Medical Centre (MUMC+), Maastricht, the Netherlands

**Keywords:** Social network structure, Social network function, Social loneliness, Emotional loneliness, COVID-19 pandemic

## Abstract

**Background:**

Loneliness is a serious public health problem. This became even more visible during the COVID-19 pandemic. Yet, the key social network aspects contributing to loneliness remain unknown. Here, we evaluated social network structure and function and associations with (moderate/severe) social and emotional loneliness in older adults.

**Methods:**

This cross-sectional study includes online questionnaire data (SaNAE cohort, August–November 2020), in independently living Dutch adults aged 40 years and older. For the separate outcomes of social and emotional loneliness, associations with structural social network aspects (e.g., network diversity - having various types of relationships, and density - network members who know each other), and functional social network aspects (informational, emotional, and practical social support) were assessed and risk estimates were adjusted for age, educational level, level or urbanization, comorbidities, and network size. Multivariable logistic regression analyses were stratified by sex.

**Results:**

Of 3396 participants (55 % men; mean age 65 years), 18 % were socially lonely which was associated with a less diverse and less dense network, living alone, feeling less connected to friends, not having a club membership, and fewer emotional supporters (men only) or informational supporters (women only). 28 % were emotionally lonely, which was associated with being socially lonely, and more exclusively online (versus in-person) contacts (men only), and fewer emotional supporters (women only).

**Conclusion:**

Network structure and function beyond the mere number of contacts is key in loneliness. Public health strategies to prevent loneliness in older adults should be sex-tailored and promote network diversity and density, club membership, informational and emotional support, and in-person contact.

## 1 Introduction

### 1.1 Loneliness: a public health problem

Loneliness is a serious public health problem. It leads to a higher risk of premature death and the onset and progression of a range of physical and mental health problems, such as cardiovascular diseases, infectious diseases, and depression [[1], [2], [3], [4]]. During the COVID-19 pandemic, social distancing measures to contain SARS-CoV-2 transmission have led to a reduction in daily contact and an increased risk of loneliness [[5], [6], [7]]. Globally, loneliness is widespread, with some countries reporting that up to one in three older people feel lonely [8]. In the Netherlands, the percentage of adults who experienced loneliness in 2022 was 49 % [9]. Loneliness also imposes a heavy financial burden on society, as it directly or indirectly results in increased healthcare costs [10,11].

### 1.2 Dimensions of loneliness

Loneliness is defined as “the unpleasant experience that occurs when a person's network of social relationships is deficient in some important way, either quantitatively or qualitatively” [12]. Weiss differentiated between social and emotional loneliness with social loneliness defined as “the absence of a broader group of contacts or an engaging social network (e.g., friends, colleagues, and people in the neighborhood)”, whereas emotional loneliness is defined as “the absence of an intimate relationship or a close emotional attachment (e.g., a partner or a best friend)” [13]. Instruments to assess loneliness usually do not distinguish between the two types, yet a validated questionnaire to assess both dimensions of loneliness is the De Jong Gierveld scale [14].

### 1.3 Social networks in relation to loneliness and preventive strategies

To address loneliness implies that we acknowledge that loneliness is related to both the structure and function of a social network, in addition to other types of relevant personal, social, and structural factors. Most previous studies assessing social network aspects in relation to loneliness have emphasized social network size (a structural social network aspect), demonstrating a link between fewer social contacts (small network size) and loneliness [15,16]. Interventions to curb loneliness will first need to identify the crucial network aspects and use these to either prevent loneliness or alleviate its consequences. Most previous interventions have focused on promoting social interactions. Yet, merely increasing the number of social relationships that a person has, is usually not effective in social and emotional loneliness [17]. Not commonly, other structural or functional network aspects are identified. Yet, a richer description of social networks based on various structural and functional aspects can be useful in the search for effective targets for the prevention of loneliness. Social networks, in which an individual is embedded, can be composed of many or few relationships, and multiple types of relationships, supports, and modes of contact [18]. Most studies examining loneliness included a limited set of such social network metrics or evaluated only ‘single’ network aspect. Though recommended is to evaluate structural and functional social network aspects jointly when assessing loneliness [[19], [20], [21]], most studies also lacked such evaluation of a combination of network aspects. As a result, in-depth knowledge of which social network aspects are most important in loneliness, in particular, emotional and social loneliness is scarce [22].

### 1.4 Structural social network aspects beyond the number of network members (network size)

Structural social network aspects include network diversity (multiple types of social relationships), network density (how well network members are connected to each other), homogeneity in terms of age and sex, geographical proximity, living alone, and mode of contact [18]. Few studies examined these other structural network aspects; family-focused (less diverse) social networks were associated with loneliness among older adults, whereas having a larger share of friends in the network was inversely associated [23,24]. People with more social network members in the neighborhood are less likely to be lonely, as these network members can meet up more easily [25,26]. Not just a lack of geographical proximity among network members, but also living alone increases the risk for loneliness [22,[27], [28], [29]].

### 1.5 Functional social network aspects

Functional social network aspects include social support from network members, such as informational, emotional, and practical support [18]. Social support may facilitate an adequate response to a possible stressful event and thereby avoiding a physical stress response or illness [30]. Lack of emotional support is associated with loneliness [31]. Also, the type of supporting relationships (family versus non-family members) was associated with the risk of emotional loneliness [32]. Social support roles have changed during the COVID-19 pandemic as people had fewer emotional or practical supporters, but also people had to depend more on their family members or neighbors for various types of support [33,34].

### 1.6 Social connectedness

During the COVID-19 pandemic, people felt less connected with family and friends [35]. Social connectedness or the subjective feeling of being embedded within a social network is important for well-being. Feeling less connected might contribute to feelings of loneliness [[36], [37], [38]].

### 1.7 Sex differences

Previous research focusing on loneliness has established sex differences. Many studies report that women are more likely to be lonely compared to men [[39], [40], [41]]. Possible explanations for this difference can be attributed to sociodemographic and health-related factors. Some studies established that practical educational level and a relatively worse health status in women in their studies resulted in reduced social interactions, and exacerbating loneliness [38,42,43]. A few studies established that men were more likely to be lonely, especially at younger age [44]. Some studies also distinguished the two dimensions of loneliness showing that women were more likely to be emotionally lonely whereas men were more likely to be socially lonely [45,46]. Moreover, sex differences are also visible in the structure and function of social networks of men and women. Women tend to have larger and more diverse social networks and receive more and different types of social support compared to men [34,47,48].

### 1.8 The present study

The Social Network Assessment in Adults and Elderly (SaNAE) study assesses social networks in relation to health. The objective of the current study is to jointly evaluate a range of structural and functional social network aspects for their association with loneliness. Specifically, the health outcomes of both social and emotional loneliness were evaluated separately among older adults during the COVID-19 pandemic. These associations are examined for men and for women, as previous studies have revealed distinct sex differences [22,34,43]. Doing so reveals possible key social network targets for preventive strategies in men and women for the identification of social and emotional loneliness and for the prevention or alleviation of loneliness.

## 2 Methods

### Ethical statement

This study was approved by the Medical Ethical Committee of the University of Maastricht (METC 2018–0698, 2019–1035, and 2020–2266). Participants gave electronic informed consent.

### 2.1 Study design and population

This cross-sectional study used data from the Dutch SaNAE cohort (www.sanae-study.nl), measured in August–November 2020 using an online questionnaire.

The SaNAE study was started in 2019 and included 5144 participants who were independent-living Dutch adults aged 40 years or older [49]. In August–November 2020, 5001 participants were invited for a follow-up questionnaire of whom 67 % (n = 3505) responded. Respondents were slightly older (mean difference 1.8 years, p < 0.001) and slightly more theoretically educated (χ^2^ = 20.884; df = 2; *p* < 0.001) compared to non-responders but did not differ in sex or network size (p > 0.05). Respondents without missing data on variables of interest in this study were included in further analyses (n = 3396).

### 2.2 Moderate or severe emotional and social loneliness (outcomes in analyses)

Loneliness was assessed using the six-item De Jong Gierveld Loneliness Scale [14]. The six-item scale can be used to measure unidimensional loneliness, but also to measure social and emotional loneliness. Three items for social loneliness included ‘There are plenty of people I can rely on when I have problems’, ‘There are many people whom I can trust completely’, and ‘There are enough people I feel close to’. The three items for emotional loneliness included ‘I experience a general sense of emptiness’, ‘I miss having people around’, and ‘I often feel rejected’. Answer categories included totally agree, agree, neutral, disagree, and totally disagree. The answer categories neutral, disagree, and totally disagree were counted for social loneliness while the answer categories neutral, agree, and totally agree were counted for emotional loneliness. A score of zero or one was defined as ‘not lonely’ whereas a score of two or three was defined as ‘(moderately/severely) lonely’ [46]. Reliability tests were performed calculating Cronbach's alpha coefficients for social and emotional loneliness items separately.

### 2.3 Social network aspects (independent variables)

Social network aspects were measured using a name-generator questionnaire. Participants were asked to provide names of family members, friends, acquaintances, and other persons who are important to them or provide social support. Additional information about network members was asked using name interpreter items. A more detailed description can be found elsewhere [34,49] and in Supplementary Table 1. We include a range of structural and functional metrics (Supplementary Table 1).

### 2.4 Statistical analyses

Descriptive analyses were used for sociodemographic characteristics and social network aspects of the study population. All analyses were performed for the outcome variables: social and emotional loneliness. All analyses were stratified by sex.

**Fig. 1:**
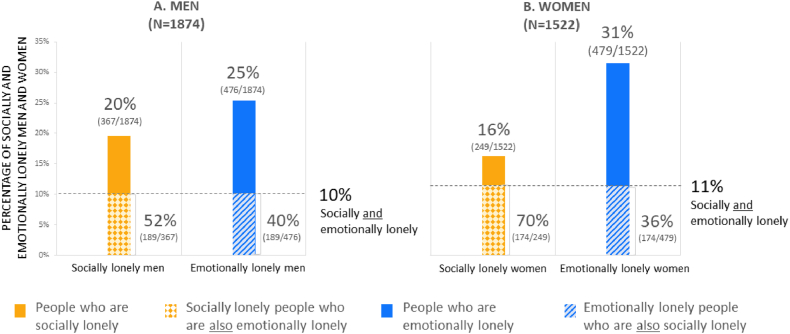
Percentages of moderate/severe social and emotional loneliness in men and women in the SaNAE study. Panel A shows percentages for men and panel B shows percentages for women.

Various multivariable logistic regression analyses models were constructed which also included the confounding variables: age, educational level, level of urbanization, and comorbidities (Type 2 Diabetes Mellitus, asthma/COPD, and cardiovascular diseases). Models (0) were created for each social network aspect separately as independent variable adjusted for confounding variables (results not presented). Models (I) were created including all social network aspects that were statistically significant from Models (0), using stepwise backward selection and also included potentially confounding variables. Models (II) additionally included social network size. Finally, for emotional loneliness as an outcome, models (III) included a variable social loneliness (as an independent determinant) since social loneliness was expected to be (in part) in the pathway between network aspects and emotional loneliness (Fig. 1).

Before the models were built, multicollinearity between social network aspects was ruled out (correlation analyses: all correlations <0.7). A *p*-value <0.05 indicated statistical significance. All analyses were performed using IBM SPSS Statistics (version 27.0).

### 2.5 Sensitivity analyses

Sensitivity analyses were performed to assess the outcomes of social and emotional loneliness when based on a different cut-off value of one and higher to reflect lonely versus not lonely (rather than the main analyses that focused on moderate/severe loneliness).

## 3 Results

### 3.1 Study population

Of all participants, 55 % were men, and 43 % of the participants had a theoretical educational level. The mean age was 65 years (Table 1).

**Table 1:** Characteristics of the SaNAE study population (n = 3396).

	Total population	Moderate/severe social loneliness			Moderate/severe emotional loneliness		
	(n = 3396)	Lonely (18 %, n = 616)	Not lonely (82 %, n = 2780)		Lonely (28 %, n = 955)	Not lonely (72 %, n = 2441)	
	% (n/N) or mean (sd)	% (n/N) or mean (sd)	% (n/N) or mean (sd)		% (n/N) or mean (sd)	% (n/N) or mean (sd)	
**Sociodemographic characteristics**							
Sex				*p* = 0.015			*p* < 0.001
Men	55 (1874/3396)	60 (367/616)	54 (1507/2780)		50 (476/955)	57 (1398/2441)	
Women	45 (1522/3396)	40 (249/616)	46 (1273/2780)		50 (479/955)	43 (1043/2441)	
Age (mean, sd)	65.2 (9.8)	65.4 (10.4)	65.1 (9.7)	*p* = 0.521	65.8 (10.3)	64.9 (9.7)	*p* = 0.017
Educational level				*p* = 0.014			*p* < 0.001
Practical	27 (915/3396)	31 (192/616)	26 (723/2780)		33 (314/955)	25 (601/2441)	
Mixed	30 (1009/3396)	30 (185/616)	30 (824/2780)		30 (289/955)	30 (720/2441)	
Theoretical	43 (1472/3396)	39 (239/616)	44 (1233/2780)		37 (352/955)	46 (1120/2441)	
Level of urbanization				p = 0.264			p = 0.080
Rural	27 (930/3396)	25 (157/616)	28 (773/2780)		25 (243/955)	29 (710/2441)	
Hardly urban	24 (810/3396)	23 (142/616)	24 (668/2780)		22 (213/955)	18 (447/2441)	
Moderately urban	19 (633/3396)	21 (131/616)	18 (502/2780)		19 (186/955)	24 (597/2441)	
Strongly or extremely urban	30 (1023/3396)	30 (186/616)	30 (837/2780)		33 (313/955)	28 (687/2441)	
**Chronic conditions**							
Type 2 Diabetes Mellitus (T2DM)	9 (300/3396)	12 (73/616)	8 (227/2780)	p = 0.004	13 (121/955)	7 (179/2441)	*p* < 0.001
Asthma/COPD	10 (338/3396)	11 (68/616)	10 (270/2780)	p = 0.320	13 (123/955)	9 (215/2441)	*p* < 0.001
Cardiovascular diseases	16 (548/3396)	19 (114/616)	16 (434/2780)	*p* = 0.077	20 (192/955)	15 (356/2441)	*p* < 0.001
Depression	5 (184/3396)	13 (80/616)	4 (104/2780)	*p* < 0.001	14 (133/955)	2 (51/2441)	*p* < 0.001
Score comorbidities				*p* < 0.001			*p* < 0.001
Zero	68 (2313/3396)	59 (364/616)	70 (1949/2780)		57 (544/955)	72 (1769/2441)	
One	22 (753/3396)	27 (169/616)	21 (584/2780)		28 (263/955)	20 (490/2441)	
Two or more	10 (330/3396)	13 (83/616)	9 (247/2780)		15 (148/955)	7 (182/2441)	

### 3.2 Moderate/severe social and emotional loneliness

Of the men, 20 % (367/1874) were socially lonely and 25 % (476/1874) were emotionally lonely. Of the women, 16 % (249/1522) were socially lonely and 31 % (479/1522) were emotionally lonely. Men were more likely to be socially lonely compared to women [aOR: 1.24, *p* < 0.05] whereas women were more likely to be emotionally lonely compared to men [aOR: 1.50, *p* < 0.001].

10 % (189/1874) of the men and 11 % (174/1522) of the women were both socially and emotionally lonely. Among the socially lonely men, 52 % (189/367) were also emotionally lonely (Fig. 1A). For socially lonely women, this was 70 % (174/249) (Fig. 1B).

### 3.3 Social network structure in people who are moderately/severely lonely

#### 3.3.1 Men

Of socially lonely men, 50 % had four or fewer network members. For 33 %, their social network was composed of only family members; 20 % had diverse social networks including family members, friends, and acquaintances who know each other (well) (Table 2). Of emotionally lonely men, 37 % had four or fewer network members. For 19 %, the social network included only family members; 30 % had diverse networks.

**Table 2:** Structural and functional social network aspects and moderate/severe social and emotional loneliness of the SaNAE study population (n = 3396).

	Moderate/severe social loneliness						Moderate/severe emotional loneliness					
	Men (n = 1874)			Women (n = 1522)			Men (n = 1874)			Women (n = 1522)		
	Lonely (n = 367)	Not lonely (n = 1507)		Lonely (n = 249)	Not lonely (n = 1273)		Lonely (n = 476)	Not lonely (n = 1398)		Lonely (n = 479)	Not lonely (n = 1043)	
	% (n) or mean (sd)	% (n) or mean (sd)		% (n) or mean (sd)	% (n) or mean (sd)		% (n) or mean (sd)	% (n) or mean (sd)		% (n) or mean (sd)	% (n) or mean (sd)	
**Social loneliness**							40 (189)	13 (178)	*p* < 0.001	36 (174)	7 (75)	*p* < 0.001
**Structural social network aspects**												
**Network size (number of network members, range 0**–**40)**	5.9 (4.9)	9.3 (6.9)	*p* < 0.001	7.6 (5.7)	12.5 (7.4)	*p* < 0.001	7.7 (6.3)	8.9 (6.7)	*p* < 0.001	9.4 (6.3)	12.7 (7.5)	*p* < 0.001
0-4 members	50 (182)	30 (445)	*p* < 0.001	37 (93)	11 (135)	*p* < 0.001	39 (185)	32 (442)	*p* = 0.003	23 (110)	11 (118)	*p* < 0.001
5-8 members	29 (108)	26 (385)		29 (73)	23 (288)		28 (132)	26 (361)		31 (149)	20 (212)	
8-13 members	12 (43)	23 (346)		20 (49)	30 (385)		18 (85)	22 (304)		24 (114)	31 (320)	
>13 members	9 (34)	22 (331)		14 (34)	37 (465)		16 (74)	21 (291)		22 (106)	38 (393)	
**Network diversity and density combined**			*p* < 0.001			*p* < 0.001			*p* = 0.010			*p* < 0.001
No family, but friends, acquaintances, or others	5 (20)	1 (19)		5 (12)	1 (12)		3 (16)	2 (23)		3 (13)	1 (11)	
Only family members	33 (121)	18 (270)		19 (48)	5 (67)		25 (117)	20 (274)		11 (52)	6 (63)	
Both family and friends, but do **not** know each other (well)	6 (23)	3 (44)		8 (21)	4 (45)		4 (20)	3 (47)		6 (30)	4 (36)	
Family, friends, acquaintances, and/or others, who do **not** know each other (well)	12 (44)	10 (146)		16 (40)	13 (171)		11 (53)	10 (137)		16 (76)	13 (135)	
Family, no friends, but others	11 (40)	9 (137)		8 (20)	5 (60)		9 (41)	10 (136)		7 (33)	5 (47)	
Both family and friends, and know each other (well)	12 (44)	15 (223)		13 (33)	13 (166)		15 (69)	14 (198)		15 (72)	12 (127)	
Family, friends, acquaintances, and/or others, and know each other (well)	20 (75)	44 (668)		30 (75)	59 (752)		34 (160)	42 (583)		42 (203)	60 (624)	
**Proportion network members of the same sex**	40.7 (28.0)	46.9 (22.4)	*p* < 0.001	58.4 (27.1)	63.7 (17.9)	p = 0.003	44.8 (24.1)	46.0 (23.6)	*p* = 0.318	62.1 (22.3)	63.2 (18.5)	p = 0.365
**Proportion network members of the same age**	40.7 (31.4)	39.5 (25.1)	*p* = 0.512	40.4 (26.2)	41.9 (20.9)	*p* = 0.385	37.6 (28.4)	40.5 (25.7)	*p* = 0.049	39.7 (23.5)	42.5 (21.0)	*p* ≤ 0.027
**Geographical proximity (proportion network members living:)**												
In the same house	27.7 (33.1)	18.3 (22.7)	*p* < 0.001	18.0 (23.6)	11.8 (14.3)	*p* < 0.001	18.5 (25.3)	20.7 (25.4)	*p* = 0.093	12.9 (18.8)	12.8 (15.1)	*p* = 0.880
Within walking distance	22.3 (27.8)	28.7 (26.1)	*p* < 0.001	26.4 (27.3)	29.4 (23.4)	*p* = 0.110	27.3 (28.1)	27.5 (26.0)	*p* = 0.869	29.7 (25.6)	28.6 (23.4)	*p* = 0.418
Less than 30 min away	31.7 (30.2)	34.8 (27.4)	p = 0.077	36.4 (27.8)	39.0 (25.8)	*p* = 0.144	35.8 (29.6)	33.7 (27.4)	*p* = 0.161	38.6 (27.9)	38.5 (25.3)	*p* = 0.949
More than 30 min away	8.5 (17.9)	10.5 (17.0)	*p* = 0.045	10.6 (18.5)	10.1 (14.7)	*p* = 0.668	9.1 (16.6)	10.4 (17.4)	*p* = 0.152	8.9 (15.5)	10.7 (15.3)	p = 0.030
Further away	9.9 (21.1)	7.7 (15.2)	p = 0.066	8.6 (17.0)	9.7 (15.7)	*p* = 0.309	9.3 (18.7)	7.7 (15.7)	*p* = 0.088	9.9 (17.0)	9.4 (15.4)	*p* = 0.583
**Mode of contact (proportion of network members:)**												
Contacted exclusively in person	61.0 (35.7)	58.3 (34.5)	p = 0.184	50.5 (36.2)	51.9 (31.6)	*p* = 0.574	55.9 (35.6)	59.8 (34.4)	*p* = 0.034	48.7 (33.4)	53.0 (31.8)	*p* = 0.018
Contacted exclusively online	20.6 (26.5)	19.6 (22.7)	*p* = 0.507	24.0 (26.3)	25.2 (21.8)	*p* = 0.487	23.6 (27.4)	18.5 (21.9)	*p* < 0.001	25.8 (24.9)	24.6 (21.5)	*p* = 0.363
Contacted both in person and online	13.3 (28.8)	16.5 (28.6)	*p* = 0.055	19.5 (30.7)	16.6 (27.2)	*p* = 0.158	14.3 (28.1)	16.4 (28.9)	*p* = 0.163	19.7 (29.9)	15.9 (26.7)	*p* = 0.017
Not contacted in the past two weeks	5.1 (13.6)	5.6 (12.7)	*p* = 0.530	6.0 (11.2)	6.4 (11.0)	*p* = 0.657	6.2 (14.3)	5.3 (12.3)	*p* = 0.204	5.8 (10.8)	6.5 (11.1)	*p* = 0.195
**Employment status**			p = 0.004			*p* = 0.062			*p* = 0.083			*p* < 0.001
Employed	28 (102)	30 (451)		32 (80)	39 (497)		27 (130)	30 (423)		29 (140)	42 (437)	
Retired	57 (209)	61 (923)		36 (89)	35 (446)		60 (285)	31 (847)		38 (180)	34 (355)	
Unemployed	14 (52)	8 (123)		31 (77)	24 (305)		12 (55)	9 (120)		32 (151)	22 (231)	
Other	1 (4)	1 (10)		1 (3)	2 (25)		1 (6)	1 (8)		2 (8)	2 (20)	
**Social participation (having a club membership)**	48 (175)	58 (874)	*p* < 0.001	45 (113)	56 (712)	p = 0.002	53 (252)	57 (797)	*p* = 0.122	51 (244)	56 (581)	*p* = 0.083
Sports club	25 (91)	32 (480)	p = 0.008	20 (50)	36 (461)	*p* < 0.001	27 (128)	32 (443)	*p* = 0.050	28 (132)	36 (379)	*p* < 0.001
Music organization	10 (36)	16 (247)	p = 0.002	10 (24)	13 (165)	p = 0.146	16 (75)	15 (208)	*p* = 0.644	11 (53)	13 (136)	*p* = 0.278
Volunteer work	10 (37)	17 (255)	p = 0.001	12 (29)	14 (178)	p = 0.325	16 (78)	15 (214)	*p* = 0.575	14 (68)	13 (139)	*p* = 0.646
Religious groups	6 (22)	4 (67)	p = 0.211	4 (9)	4 (50)	p = 0.815	7 (33)	4 (56)	*p* = 0.010	4 (19)	4 (40)	*p* = 0.902
Talking groups	3 (11)	3 (48)	p = 0.853	4 (11)	3 (39)	p = 0.273	5 (22)	3 (37)	*p* = 0.033	4 (18)	3 (32)	*p* = 0.483
Other	18 (67)	18 (277)	p = 0.956	20 (50)	19 (240)	p = 0.652	17 (81)	19 (263)	*p* = 0.382	21 (100)	18 (190)	*p* = 0.220
**Functional social network aspects**												
**Number of informational supporters**	2.8 (3.3)	4.3 (4.7)	*p* < 0.001	3.4 (3.8)	6.2 (5.6)	*p* < 0.001	3.6 (4.3)	4.1 (4.5)	*p* = 0.035	4.6 (4.4)	6.2 (5.8)	*p* < 0.001
**Number of emotional supporters**	3.4 (3.5)	5.7 (5.4)	*p* < 0.001	4.8 (4.6)	7.8 (5.9)	*p* < 0.001	4.7 (4.9)	5.5 (5.3)	*p* = 0.003	5.7 (4.5)	8.1 (6.2)	*p* < 0.001
**Number of practical supporters**	1.4 (1.8)	1.9 (2.2)	*p* < 0.001	1.3 (1.5)	2.0 (2.0)	*p* < 0.001	1.7 (2.0)	1.8 (2.2)	*p* = 0.396	1.8 (2.1)	2.0 (1.9)	*p* = 0.059
**Connectedness with family**^a^			*p* = 0.002			*p* < 0.001			*p* < 0.001			*p* < 0.001
Less connected	16 (57)	10 (149)		23 (56)	9 (118)		19 (900)	8 (116)		21 (101)	7 (73)	
Unchanged	74 (272)	76 (1140)		65 (163)	71 (905)		67 (320)	78 (1092)		63 (303)	73 (765)	
More connected	10 (38)	15 (218)		12 (30)	20 (250)		14 (66)	14 (190)		16 (75)	20 (205)	
**Connectedness with friends**^a^			*p* < 0.001			*p* < 0.001			*p* < 0.001			*p* < 0.001
Less connected	22 (82)	14 (217)		29 (72)	15 (193)		28 (135)	12 (164)		31 (148)	11 (117)	
Unchanged	73 (267)	78 (1173)		66 (163)	73 (923)		63 (300)	82 (1140)		60 (285)	77 (801)	
More connected	5 (18)	8 (117)		6 (14)	12 (157)		9 (41)	7 (94)		10 (46)	12 (125)	
**Connectedness with people at work**^a^			*p* = 0.863			p = 0.003			*p* < 0.001			*p* < 0.001
Less connected	26 (96)	25 (381)		37 (91)	26 (336)		36 (170)	22 (307)		38 (183)	23 (244)	
Unchanged	71 (262)	73 (1094)		60 (149)	67 (858)		62 (293)	76 (1063)		57 (271)	71 (736)	
More connected	3 (9)	2 (32)		4 (9)	6 (79)		3 (13)	2 (28)		5 (25)	6 (63)	
**Connectedness with neighbors**^a^			*p* = 0.015			*p* < 0.001			*p* < 0.001			*p* < 0.001
Less connected	14 (52)	10 (148)		25 (63)	12 (156)		20 (93)	8 (107)		25 (119)	10 (100)	
Unchanged	77 (284)	84 (1262)		68 (169)	79 (1001)		72 (344)	86 (1202)		68 (324)	81 (846)	
More connected	8 (31)	6 (97)		7 (17)	9 (116)		8 (39)	6 (89)		8 (36)	9 (97)	
**Connectedness with my city**^a^			*p* = 0.789			*p* = 0.019			*p* < 0.001			*p* < 0.001
Less connected	20 (75)	20 (297)		31 (78)	23 (292)		31 (148)	16 (224)		38 (184)	18 (186)	
Unchanged	77 (282)	77 (1159)		65 (162)	73 (927)		65 (311)	81 (1130)		59 (280)	78 (809)	
More connected	3 (10)	3 (51)		4 (9)	4 (54)		4 (17)	3 (44)		3 (15)	5 (48)	
**Connectedness with my country**^a^			*p* = 0.019			*p* < 0.001			*p* < 0.001			*p* < 0.001
Less connected	21 (77)	15 (226)		29 (71)	17 (212)		25 (120)	13 (183)		31 (149)	13 (134)	
Unchanged	73 (268)	79 (1193)		66 (164)	75 (959)		37 (320)	82 (1141)		62 (299)	79 (824)	
More connected	6 (22)	6 (88)		6 (14)	8 (102)		8 (36)	5 (74)		7 (31)	8 (85)	
**Connectedness with the world**^a^			*p* = 0.015			*p* = 0.003			*p* < 0.001			*p* < 0.001
Less connected	25 (93)	19 (290)		30 (75)	21 (266)		29 (136)	18 (247)		33 (159)	17 (182)	
Unchanged	71 (261)	75 (1133)		66 (164)	72 (921)		66 (314)	77 (1080)		61 (290)	76 (795)	
More connected	4 (13)	6 (84)		4 (10)	7 (86)		6 (26)	5 (71)		6 (30)	6 (66)	

a Compared to pre-COVID-19 times.

#### 3.3.2 Women

Of socially lonely women, 39 % had four or fewer network members. For 25 %, their social network was composed of only family members; 34 % had diverse social networks including family members, friends, and acquaintances who know each other (well) (Table 2). Of emotionally lonely women, 23 % had four or fewer network members. For 11 %, the social network included only family members; 42 % had diverse networks.

### 3.4 Social network aspects associated with moderate/severe social loneliness

#### 3.4.1 Men

In model I, independently associated with social loneliness was having less diverse and less dense social networks, a larger proportion of network members living in the same house or living far away, living alone, not participating in work, not being a member of a music organization, having fewer emotional supporters and feeling less connected with friends, and feeling more connected with neighbors (Table 3). After adding network size (model II), all these social network aspects, except social network size and not being a member of a music organization, remained associated.

**Table 3:** Multivariable logistic regression analyses between structural and functional social network aspects and moderate/severe social loneliness.

Moderate/severe social loneliness	Model I		Model II	
	**Men**	**Women**	**Men**	**Women**
	**OR (95 % CI)**	**OR (95 % CI)**	**OR (95 % CI)**	**OR (95 % CI)**
**Structural social network aspects**				
**Network size (number of network members)**	Not assessed	Not assessed		
0-4 members			1.12 (0.60–2.08)	**3.77 (1.81**–7.82) ***
5-8 members			1.28 (0.75–2.17)	**1.93 (1.06**–3.52) *
8-13 members			0.90 (0.53–1.51)	1.34 (0.79–2.28)
>13 members			Ref	Ref
**Network diversity and density combined**				
No family, but friends, acquaintances, or others	**4.68 (2.26**–9.71) ***	**4.42 (1.73**–11.30) **	**4.67 (2.21**–9.87) ***	**2.89 (1.08**–7.72) *
Only family	**2.03 (1.34**–3.06) ***	**3.50 (2.02**–6.06) ***	**1.97 (1.27**–3.04) **	**2.29 (1.27**–4.14) **
Both family and friends, but do not know each other (well)	**3.12 (1.73**–5.64) ***	**2.72 (1.45**–5.10) **	**2.89 (1.58**–5.28) ***	**2.18 (1.14**–4.18) *
Family, friends, acquaintances, and/or others, but do not know each other (well)	**2.20 (1.43**–3.38) ***	**1.96 (1.26**–3.05) **	**2.18 (1.41**–3.36) ***	**2.05 (1.31**–3.22) **
Family, no friends, but others	**1.86 (1.18**–2.94) **	**2.09 (1.14**–3.84) *	**1.78 (1.12**–2.84) *	1.75 (0.94–3.26) #
Both family and friends, and know each other (well)	1.38 (0.90–2.11)	1.31 (0.81–2.11)	1.29 (0.83–2.01)	1.14 (0.69–1.86)
Family, friends, acquaintances, and/or others, and they know each other (well)	Ref	Ref	Ref	Ref
**Proximity (proportion network members living:)**				
In the same house	**2.50 (1.46**–4.29) ***	2.08 (0.80–5.40)	**2.51 (1.44**–4.37) **	1.32 (0.50–3.53)
Further away	**2.26 (1.13**–4.52) *	0.73 (0.27–1.93)	**2.25 (1.13**–4.49) *	0.79 (0.30–2.12)
**Living alone**	**1.77 (1.28**–2.46) ***	**1.87 (1.28**–2.72) **	**1.75 (1.26**–2.43) **	**1.76 (1.20**–2.58) **
**Employment status**				
Employed	Ref	Ref	Ref	Ref
Retired	0.80 (0.53–1.21)	1.45 (0.88–2.40)	0.82 (0.54–1.24)	1.39 (0.83–2.32)
Unemployed	**1.65 (1.07**–2.56) *	1.38 (0.91–2.09)	**1.65 (1.07**–2.56) *	1.31 (0.86–2.00)
Other	1.08 (0.29–1.39)	0.65 (0.17–2.44)	1.11 (0.30–4.11)	0.68 (0.18–2.55)
**No membership of a sports club**	1.04 (0.78–1.39)	**1.86 (1.30**–2.67) ***	1.04 (0.78–1.39)	**1.85 (1.29**–2.67) ***
**No membership of a music organization**	**1.50 (1.01**–2.22) *	1.14 (0.70–1.87)	1.47 (0.99–2.19) #	1.09 (0.66–1.793)
**Functional social network aspects**				
**Fewer informational supporters**	0.99 (0.95–1.03)	**1.08 (1.02**–1.14) **	0.99 (0.94–1.03)	**1.07 (1.01**–1.13) **
**Fewer emotional supporters**	**1.08 (1.03**–1.12) ***	1.02 (0.97–1.07)	**1.07 (1.02**–**1.12)** **	0.98 (0.93–1.03)
**Connectedness with family**^a^				
Less connected	1.15 (0.76–1.76)	**1.71 (1.06**–2.75) *	1.16 (0.76–1.78)	**1.71 (1.05**–2.77) *
Unchanged	Ref	Ref	Ref	Ref
More connected	0.91 (0.57–1.45)	0.92 (0.55–1.54)	0.92 (0.58–1.47)	0.94 (0.56–1.58)
**Connectedness with friends**^a^				
Less connected	**1.55 (1.07**–2.25) *	1.29 (0.85–1.97)	**1.54 (1.06**–**2.24)** *	1.26 (0.83–1.94)
Unchanged	Ref	Ref	Ref	Ref
More connected	0.98 (0.52–1.87)	0.65 (0.32–1.33)	0.97 (0.51–1.85)	0.64 (0.31–1.32)
**Connectedness with neighbors**^a^				
Less connected	1.25 (0.85–1.86)	**1.79 (1.20**–2.68) **	1.24 (0.84–1.85)	**1.77 (1.18**–2.66) **
Unchanged	Ref	Ref	Ref	Ref
More connected	**1.79 (1.11**–2.89) *	1.42 (0.78–2.59)	**1.78 (1.10**–**2.87)** *	1.45 (0.80–2.66)

Model I: adjusted for age, educational level, level of urbanization, type 2 Diabetes Mellitus, asthma/COPD, and cardiovascular diseases.

Model II: adjusted for age, educational level, level of urbanization, type 2 Diabetes Mellitus, asthma/COPD, cardiovascular diseases, and network size.

OR: odds ratio, 95%CI: 95 % confidence interval. ^#^*p* < 0.1, *p < 0.05, **p < 0.01, ***p < 0.001.

a Compared to pre-COVID-19 times.

#### 3.4.2 Women

In model I, independently associated with social loneliness were having less diverse and less dense social networks, living alone, not being a member of a sports club, having fewer informational supporters, and feeling less connected with family and neighbors. After adding network size (model II), all these social network aspects remained associated. Smaller social network size was also associated with social loneliness (Table 3).

### 3.5 Social network aspects associated with moderate/severe emotional loneliness

#### 3.5.1 Men

In model I, independently associated with emotional loneliness were having less diverse and less dense social networks, contacting a larger proportion of network members exclusively online, living alone, membership of a religious group, feeling less connected to friends, people from work and the city, and feeling more connected with friends, and the country. After adding network size (model II), all these social network aspects remained associated, except for network diversity and density. After adding social loneliness (model III), all social network aspects remained associated, except feeling more connected to the country. Social loneliness was also associated with emotional loneliness (Table 4).

**Table 4:** Multivariable logistic regression analyses between structural and functional social network aspects and moderate/severe emotional loneliness.

Moderate/severe emotional loneliness	Model I		Model II		Model III	
	**Men**	**Women**	**Men**	**Women**	**Men**	**Women**
	**OR (95 % CI)**	**OR (95 % CI)**	**OR (95 % CI)**	**OR (95 % CI)**	**OR (95 % CI)**	**OR (95 % CI)**
**Structural social network aspects**						
**Social loneliness**	Not assessed	Not assessed	Not assessed	Not assessed	**4.37 (3.30**–5.79) ***	**6.13 (4.34**–8.67) ***
**Network size**	Not assessed	Not assessed				
0-4 members			1.15 (0.69–1.94)	1.65 (0.95–2.84) #	1.10 (0.64–1.89)	1.12 (0.63–2.01)
5-8 members			1.15 (0.73–1.80)	**1.61 (1.06**–2.44) *	1.11 (0.70–1.77)	1.37 (0.89–2.12)
8-13 members			1.12 (0.74–1.68)	1.00 (0.69–1.43)	1.18 (0.78–1.81)	0.93 (0.64–1.35)
>13 members			Ref	Ref	Ref	Ref
**Network diversity and density combined**						
No family, but friends, acquaintances, or others	1.60 (0.77–3.36)	1.60 (0.64–3.96)	1.57 (0.74–3.37)	1.33 (0.52–3.42)	0.90 (0.39–2.08)	0.85 (0.30–2.38)
Only family	**1.46 (1.04**–2.06) *	**1.86 (1.16**–2.99) *	1.42 (0.96–2.11) #	1.45 (0.85–2.49)	1.14 (0.75–1.72)	1.09 (0.61–1.95)
Both family and friends, but do not know each other (well)	1.18 (0.63–2.21)	1.54 (0.86–2.76)	1.14 (0.60–2.16)	1.29 (0.71–2.34)	0.80 (0.40–1.58)	1.03 (0.54–1.98)
Family, friends, acquaintances, and/or others, but do not know each other (well)	1.05 (0.70–1.57)	**1.53 (1.07**–2.18) *	1.05 (0.70–1.57)	**1.53 (1.07**–2.19) *	0.86 (0.56–1.31)	1.31 (0.90–1.92)
Family, no friends, but others	1.05 (0.68–1.63)	**1.70 (0.99**–**2.90) #**	1.03 (0.66–1.60)	1.49 (0.86–2.58)	0.86 (0.54–1.37)	1.31 (0.73–2.37)
Both family and friends, and know each other (well)	1.22 (0.85–1.76)	1.20 (0.83–1.76)	1.19 (0.81–1.74)	1.07 (0.73–1.59)	1.11 (0.75–1.64)	1.06 (0.71–1.60)
Family, friends, acquaintances, and/or others, and they know each other (well)	Ref	Ref	Ref	Ref	Ref	Ref
**Mode of contact**						
In person contact	1.16 (0.78–1.73)	0.69 (0.45–1.07) #	1.16 (0.77–1.71)	0.71 (0.46–1.11) #	1.06 (0.70–1.61)	0.71 (0.45–1.13) #
Online contact	**2.15 (1.24**–3.73) **	0.99 (0.53–1.84)	**2.15 (1.24**–3.73) **	1.04 (0.56–1.94)	**1.99 (1.12**–3.55) *	1.00 (0.52–1.92)
**Living alone**	**3.32 (2.51**–4.39) ***	**2.08 (1.57**–2.76) ***	**3.31 (2.50**–4.38) ***	**2.08 (1.57**–2.77) ***	**3.26 (2.43**–4.37) ***	**1.95 (1.44**–2.63) ***
**Employment status**						
Employed	Ref	Ref	Ref	Ref	Ref	Ref
Retired	0.91 (0.62–1.34)	1.25 (0.83–1.87)	0.91 (0.62–1.34)	1.19 (0.79–1.79)	0.97 (0.65–1.46)	1.13 (0.74–1.73)
Unemployed	1.05 (0.68–1.62)	**1.67 (1.19**–2.35) **	1.06 (0.69–1.62)	**1.61 (1.14**–2.27) **	0.95 (0.61–1.49)	**1.57 (1.10**–2.25) *
Other	1.82 (0.53–6.30)	1.14 (0.45–2.92)	1.83 (0.53–6.35)	1.18 (0.46–3.04)	1.97 (0.58–6.76)	1.28 (0.49–3.34)
**Membership of religious groups**	**1.87 (1.14**–3.09) *	0.94 (0.50–1.74)	**1.88 (1.14**–3.09) **	0.96 (0.51–1.79)	**1.78 (1.06**–2.99) *	0.88 (0.46–1.72)
**Functional social network aspects**						
**Fewer emotional supporters**	1.02 (0.99–1.04)	**1.06 (1.03**–1.09) ***	1.01 (0.98–1.04)	**1.04 (1.01**–1.08) *	1.00 (0.97–1.03)	**1.04 (1.01**–1.08) *
**Connectedness with friends**^a^						
Less connected	**2.49 (1.83**–3.38) ***	**2.79 (2.01**–3.86) ***	**2.49 (1.83**–3.39) ***	**2.75 (1.99**–3.82) ***	**2.39 (1.74**–3.30) ***	**2.69 (1.91**–3.80) ***
Unchanged	Ref	Ref	Ref	Ref	Ref	Ref
More connected	**1.58 (1.01**–2.47) *	1.26 (0.81–1.95)	**1.59 (1.02**–2.49) *	1.27 (0.82–1.98)	**1.71 (1.08**–2.71) *	1.40 (0.89–2.20)
**Connectedness with people from work**^a^						
Less connected	**1.33 (1.02**–1.74) *	**1.37 (1.03**–1.81) *	**1.34 (1.02**–1.75) *	**1.38 (1.04**–1.83) **	**1.40(1.06**–1.86) *	**1.35 (1.00**–1.81) *
Unchanged	Ref	Ref	Ref	Ref	Ref	Ref
More connected	1.84 (0.86–3.94)	**1.83 (1.04**–3.21) *	1.84 (0.86–3.93)	1.76 (1.00–3.11) #	1.66 (0.75–3.68)	**1.90 (1.06**–3.42) *
**Connectedness with neighbors**^a^						
Less connected	1.44 (0.99–2.10) #	1.40 (0.97–2.02) #	1.44 (0.98–2.10) #	1.37 (0.95–1.99) #	1.39 (0.94–2.05)	1.21 (0.82–1.79)
Unchanged	ref	ref	Ref	ref	ref	ref
More connected	1.37 (0.87–2.16)	1.06 (0.66–1.69)	1.38 (0.87–2.16)	1.08 (0.68–1.74)	1.22 (0.76–1.96)	1.03 (0.63–1.67)
**Connectedness with city**^a^						
Less connected	**1.50 (1.02**–2.19) *	**1.46 (1.01**–2.12) *	**1.50 (1.03**–2.19) **	**1.48 (1.02**–2.15) *	**1.79 (1.21**–2.67) *	**1.64 (1.11**–2.42) *
Unchanged	Ref	Ref	Ref	Ref	Ref	Ref
More connected	0.69 (0.32–1.48)	0.67 (0.30–1.48)	0.69 (0.32–1.48)	0.68 (0.31–1.51)	0.79 (0.36–1.73)	0.66 (0.29–1.52)
**Connectedness with country**^a^						
Less connected	1.32 (0.89–1.95)	1.46 (1.00–2.15) #	1.31 (0.88–1.94)	1.45 (0.99–2.14) #	1.11 (0.74–1.67)	1.40 (0.93–2.10)
Unchanged	Ref	Ref	Ref	Ref	Ref	Ref
More connected	**1.88 (1.08**–3.26) *	1.04 (0.59–1.85)	**1.89 (1.08**–3.28) *	1.04 (0.58–1.84)	1.74 (0.97–3.09) #	1.07 (0.59–1.93)

Model I: adjusted for age, educational level, level of urbanization, type 2 Diabetes Mellitus, asthma/COPD, cardiovascular diseases.

Model II: adjusted for age, educational level, level of urbanization, type 2 Diabetes Mellitus, asthma/COPD, cardiovascular diseases, and network size.

Model III: adjusted for age, educational level, level of urbanization, type 2 Diabetes Mellitus, asthma/COPD, cardiovascular diseases, network size, and social loneliness.

OR: odds ratio, 95%CI: 95 % confidence interval. ^#^*p* < 0.1, *p < 0.05, **p < 0.01, ***p < 0.001.

a Compared to pre-COVID-19 times.

#### 3.5.2 Women

In model I, independently associated with emotional loneliness were less diverse and less dense social networks, living alone, not participating in work, having fewer emotional supporters, and feeling less connected with friends, people from work, and the city, and feeling more connected with people from work. After adding network size (model II), all these social network aspects remained associated except feeling more connected to people from work. Smaller network size was also associated. After adding social loneliness (model III), all social network aspects remained associated except network size and diversity and density.

**Fig. 2:**
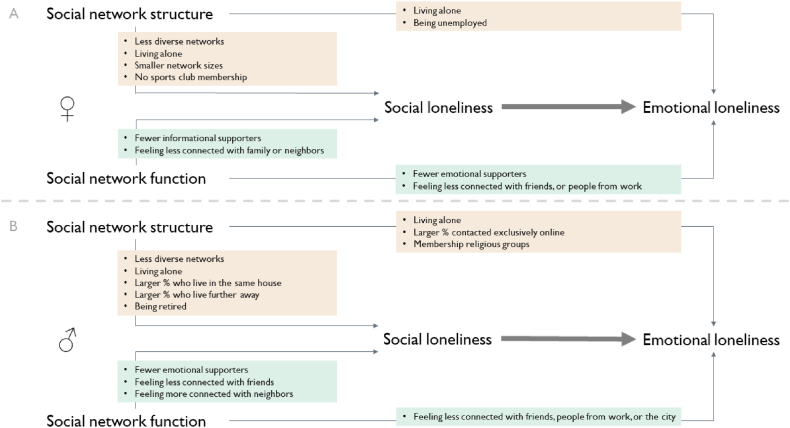
Overview of associations between social network aspects and moderate/severe social and emotional loneliness among men and women in the SaNAE study. Panel A shows associations for women and panel B shows association for men.

A visual overview of all associations between social networks and social and emotional loneliness is available in Fig. 2A,B.

Sensitivity analyses showed similar results for the observed associated network aspects, when using another cut-off value (≥1) for both social and emotional loneliness (Supplementary Tables 2 and 3).

## 4 Discussion

This detailed evaluation of loneliness in Dutch adults aged 40 years and older, uniquely evaluated the structure and function of social networks and did so in relation to social loneliness as well as emotional loneliness. Of men, 20 % and 25 % were (moderately/severely) socially and emotionally lonely, respectively. Of women, 16 % and 32 % were (moderately/severely) socially and emotionally lonely, respectively. More than half of the people who were socially lonely, also were emotionally lonely. A sizeable proportion (19%–24 %) ‘only’ were emotionally lonely. By evaluating a wide range of structural and functional social network aspects, jointly, this study was able to identify the most important social network aspects for men and women, and for the two dimensions of loneliness. These insights can be further used to address of relevant social network aspects and strategies to prevent or alleviate loneliness.

Loneliness is a profoundly unpleasant experience and not merely defined by either a structural or functional social network aspect. This experience can certainly be more than ‘just’ the lack of the number of relationships or lack of social support. Striking differences were observed for social or emotional loneliness in this respect as well. Men and women in the current study were more (moderately/severely) socially lonely when their social network was less diverse (e.g., family-centered), in line with previous studies [15,16,23,24], or less dense (friends and family clustered less). Also, a smaller network size was associated with social loneliness (women only) in line with previous studies [31]. Further, men and women were more likely to be socially lonely when they lived alone. Living alone is a well-known factor in loneliness and other adverse health outcomes [[27], [28], [29],^40^]. Also important in social loneliness were feeling less connected to friends, fewer emotional supporters (for men only), fewer informational supporters (for women only), and not having a club membership.

Men and women were (moderately/severely) lonely when they also felt socially lonely, when they lived alone, when a larger proportion of their network members was contacted exclusively online (for men only), or when having fewer emotional supporters (for women only). Various metrics have been shown to be important in previous studies [22], while this study thus also revealed novel insights.

Important to highlight is that less diverse and less dense social networks likely posed a risk for social or emotional loneliness in men and women, regardless of the number of social relationships a person has. This has implications for assessing social network structure in relation to health, indicating the importance of taking network diversity and density into account. Also notable was that women who had fewer informational supporters and men who had fewer emotional supporters were more likely to be socially lonely, indicating that men and women may have different needs for social support from their social network. Previous studies have already established sex differences in the type of social support and type of social relationships providing support, with women receiving more emotional support from different types of relationships [34,47]. Finally, online contact (for men) was detrimental to emotional loneliness, highlighting the value of in-person contact which was also observed in a qualitative study [50].

Physical contact with social network members was reduced and (temporarily) replaced with online contact during the COVID-19 pandemic [34]. Especially those who live alone were affected by these measures since online contact among individuals who live alone was in previous studies with negative emotions and loneliness [51,52]. Online contact might not be a full substitute for in-person contact [53] and it has been demonstrated to decrease the level of feeling connected to others, which in turn negatively impacts health [35].

Social participation was also linked to social loneliness, as women in the current study who did not have a sports club membership more often were lonely. Previous studies have reported that women who exercise with others may do so for motivation and social conviviality [54], and exercising together stimulates them to exercise more frequently [[55], [56], [57]]. Men in this study who were members of religious groups were more likely to be emotionally lonely, and the reasons are unknown and could be a topic of further study.

Interestingly, men who were retired were more likely to be socially lonely and women who were unemployed were more likely to emotionally lonely. A possible explanation could be that women receive more social support from coworkers compared to men, and are more likely to consider their coworkers as their friends [58].

### 4.1 Implications

To curb trends in loneliness is a key public health priority. This means to better identify relevant social network aspects in loneliness and to better use these insights for designing strategies to prevent or alleviate loneliness. The current study provides insight into possible targets for the identification of loneliness and strategies to prevent or to alleviate loneliness. These results stress the importance of looking beyond the number of social relationships and consider the rich variety of social network aspects. Moreover, this rich variety of social network aspects for social and emotional loneliness differ for men and women. Therefore, sex differences should be taken into account in preventive strategies.

Examples for better identification include a small network size, but also less diverse relationships as well as employment status and living alone, factors that may serve as indicators to recognize possible loneliness. Targeting social loneliness may also in part target emotional loneliness, but not completely as different social network aspects are important for both constructs. In line with the work of Holt-Lunstad, we suggest that future research should focus on the development of social connection guidelines including the social network aspects identified in the current study [59].

Examples for preventive strategies focused on network structure could be to help people to expand their network by adding new network members to also create a more diverse social network, and by strengthening connections between social network members to increase density. Club memberships (e.g., sports or music organizations) should be promoted and facilitated to enable people to meet new people in leisure activities. Strategies should further build on strengthening social network function for social support in existing social network members and relevant support in new members. This means that strategies should primarily focus on the social environment rather than only the individual living in it.

### 4.2 Strengths and limitations

A strength of the current study is that we used a large cohort study that uniquely and jointly assessed various structural and functional social network aspects in men and women, in combination with health outcomes such as social and emotional loneliness. To assess the social network aspects, a name generator questionnaire with name interpreter items was used, which is a useful method for measuring social networks in online surveys extracting large and diverse networks [60]. Furthermore, we evaluated the social network aspects separately for social and emotional loneliness and thereby identified which aspects are most important for each type of loneliness. Some limitations should be mentioned. Different answer categories for the De Jong Gierveld scale were used. Instead of three answer categories: ‘yes’, ‘more or less’, and ‘no’, a five-Likert scale was used “totally agree – agree – neutral – disagree – totally disagree”. Reliability tests to determine internal consistency reliability for social and emotional loneliness items were performed. Items showed high internal consistency. (social loneliness items: α = 0.911, and emotional loneliness items: α = 0.794). Furthermore, due to the cross-sectional design of the current study, no conclusions can be drawn on the causality of the effects.

## 5 Conclusion

Our current study assessed structural and functional social network aspects associated with (moderately/severely) social and emotional loneliness for men and women separately and established that diverse and dense social networks and emotional and informational social support are key factors associated with social and emotional loneliness. Other relevant determinants were geographical proximity, mode of contact, club membership, employment status, living alone, and social cohesion. Preventive strategies to alleviate or prevent loneliness should focus on both structural and functional network aspects, need to look beyond the number of social relationships and promote diverse and supporting social relationships.

## Data availability statement

The dataset supporting the conclusions of this article is available upon request. Data contains potentially identifying and sensitive information of respondents. Due to the General Data Protection Regulation, it is not allowed to distribute or share any personal data that can be traced back (direct or indirect) to an individual. Moreover, publicly sharing the data would not be in accordance with participant consent for this study.

## CRediT authorship contribution statement

**Lisanne CJ. Steijvers:** Writing – original draft, Visualization, Methodology, Formal analysis, Conceptualization. **Stephanie Brinkhues:** Writing – review & editing, Visualization, Supervision, Methodology, Formal analysis, Conceptualization. **Bianca Suanet:** Writing – review & editing. **Mandy MN. Stijnen:** Writing – review & editing. **Christian JPA. Hoebe:** Writing – review & editing, Supervision. **Nicole HTM. Dukers-Muijrers:** Writing – review & editing, Visualization, Supervision, Methodology, Formal analysis, Conceptualization.

## Declaration of competing interest

The authors declare that they have no known competing financial interests or personal relationships that could have appeared to influence the work reported in this paper.
